# Study on the Purcell Effect and Photoluminescence Properties of Gold–Titanium Dioxide Quasiperiodic Multilayers and Cavities

**DOI:** 10.3390/nano15191502

**Published:** 2025-10-01

**Authors:** Guangfa He, Changjun Min, Ling Li, Xiaocong Yuan

**Affiliations:** Nanophotonics Research Center, Institute of Microscale Optoelectronics & State Key Laboratory of Radio Frequency Heterogeneous Integration, Shenzhen University, Shenzhen 518060, China; 2200493002@email.szu.edu.cn (G.H.); cjmin@szu.edu.cn (C.M.);

**Keywords:** Purcell factor, quasiperiodic multilayer, multilayer cavity

## Abstract

This work studies the Purcell effect of two quasiperiodic multilayers of gold and titanium dioxide following the Thue–Morse and Fibonacci sequence, respectively. We systematically investigated the impacts of polarization direction, dipole height, and wavelength on the Purcell factor. Additionally, we compared the normalized field distribution profiles across all multilayer structures. Concurrently, under varying polarizations, we computed the radiative part of the Purcell factor, photoluminescence at the reflection and transmission side of multilayers, respectively. Our findings indicate that under near-field excitation conditions, the Purcell factor is predominantly governed by its non-radiative component rather than the radiative one. We attribute the observed discrepancies in the Purcell factor to variations in the intensity and spatial distribution of non-radiative losses within the metallic components of the multilayers. This mechanism provides a robust physical foundation for exploring and extending the applications of photonic quasicrystals in the modulation of nanoscale light–matter interactions. Furthermore, we examined cavities constructed from symmetric multilayers. Under *z*-polarization and long-wavelength conditions, the cavity effect was observed to enhance the radiative part of the Purcell factor, thereby further boosting spontaneous emission efficiency. This work offers novel insights into the design of semiconductor devices with improved quantum emission efficiency and photoluminescence.

## 1. Introduction

Quasicrystals represent an important intermediate state of matter between crystalline and disordered solids [1]. In the case of disordered effect, the discovery of Anderson localization of electrons has spawned a broad spectrum of research on wave localization, including spin-waves, acoustic waves, and ultracold atoms in random trapping potentials [2,3]. A surge of works introduced randomness or disorder to photonic structures to isolate wave localization from other complicating interactions prevailing in electron systems [4,5,6,7,8,9]. In stark contrast to electrons, there is a lack of direct interactions among photons transporting through photonic media. Photonic quasicrystals (PQCs) became essential to study many novel interactions between electromagnetic waves with artificial photonic materials, such as realizing electromagnetic wave localization [6,7,8,9,10,11,12,13], enhancing emission in light-emitting structures, and compressing ultrafast laser pulses [14,15]. Fundamentally, it is the property of disorder without periodicity in PQCs that renders the quasi-localization nature of the band edge state—an essential factor responsible for all the interesting phenomena observed. Moreover, the co-existence of short-range disorder and long-range wave correlation distinguishes PQCs from both photonic crystals and amorphous glasses [7,8,9,16]. Compared with the vast amount of works on passive properties of PQCs, studies that probe the active effect of PQCs on external emitters remain scarce.

There have been tremendous research interests and efforts in engineering photonic local density of states (LDOS) with periodic multilayer metamaterials to enhance spontaneous emission [17,18,19,20,21,22,23]. The LDOS enhancement effect of multilayer metamaterials contributes to a plethora of endeavors, such as enhancing single photon emission from multilayer hexagonal boron nitride [24], boosting slow terahertz emission with multilayer graphene [25,26], controlling chiral emission of electric dipoles [27], increasing both modulation speed and light efficiency of light-emitting diodes [28], and enhancing light transmission performance in photonic crystals by embedding graphene [29]. Dielectric PQCs were studied for their spontaneous emission enhancement effects [15,30]. However, research on the application of quasiperiodic metal–dielectric multilayers in spontaneous emission enhancement has only emerged in recent years [31,32,33,34,35,36,37,38,39,40].

In this work, we study the Purcell effect of a Fibonacci multilayer (FM) and a Thue-Morse multilayer (THM) consisting of gold and titanium dioxide (TiO_2_) layers. Their Purcell effects are compared with a periodic multilayer (PM) theoretically. Furthermore, we constructed cavities with these multilayers, analyzed the localization characteristics of the electric field magnitude distribution in both the multilayers and the cavities, and systematically investigated their photoluminescent properties. This study aims to address two key research questions: (1) What is the primary factor accounting for the discrepancies in Purcell factors (PFs) among these multilayers? (2) What novel characteristics are exhibited by the photoluminescence (PL) of the cavities? Meanwhile, there remains a notable lack of systematic comparison concerning how metal–dielectric multilayers with different periodicities influence the enhancement of spontaneous emission. To address this gap, we have conducted an extensive and comprehensive study in terms of the effects of wavelength, polarization state, dipole height, and periodicity. We found that the primary contribution to the PF of the multilayers stems from non-radiative losses. More importantly, compared with composite plasmonic antennas [41], quasiperiodic multilayers, and Bragg reflectors [34], the quasiperiodic multilayer cavities studied in our work offer greater advantages in enhancing spontaneous emission through two distinct pathways: 1. Non-radiative metallic losses within the multilayer walls; 2. Radiative resonance induced by the cavity effect. This advantage enables the cavities to sustain a high PF across a broad wavelength range. Our work not only delivers additional theoretical support for regulating the PF of quasiperiodic multilayers but also provides insights into the future design of cavity devices tailored to enhance spontaneous emission.

## 2. Structure and Methods

**Figure 1:**
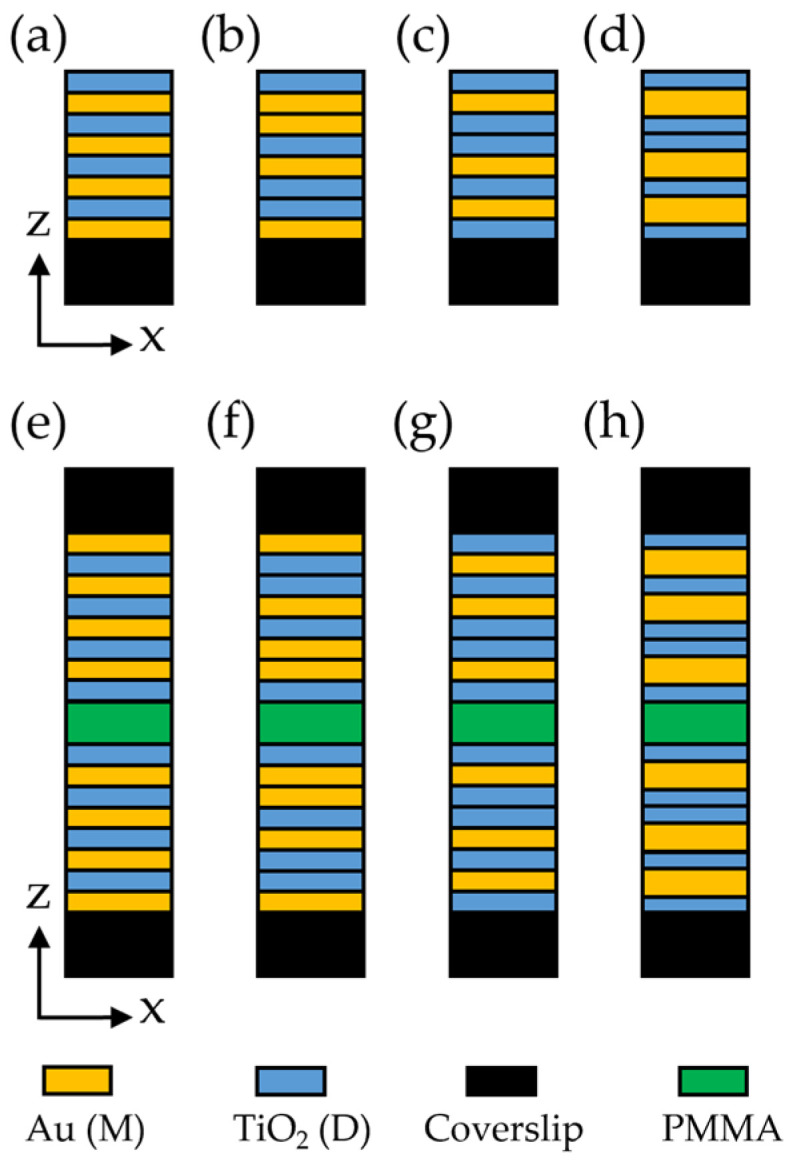
Schematic illustrations of multilayers and cavities: (**a**) PM, (**b**) THM, (**c**) FM, (**d**) FM*, (**e**) PM cavity, (**f**) THM cavity, (**g**) FM cavity, and (**h**) FM* cavity. The gold, titanium dioxide, coverslip substrate, and PMMA are denoted in orange, blue, black, and green, respectively. The Au layer and TiO_2_ layer in (**a**–**c**,**e**–**g**) each have a thickness of 10 nm, whereas those in (**d**,**h**) have thicknesses of 13.33 nm and 8 nm, respectively. The PMMA layer in the cavity has a thickness of 20 nm.

Figure 1a–c depict the schematic illustration images of the PM, THM, and FM, in which the orange and blue regions are gold [42] and TiO_2_ [43] layers, respectively. To reveal the lattice order-induced difference in the Purcell effect from these one-dimensional photonic structures, we keep identical the overall structure thicknesses of these multilayers. These multilayers start with a dielectric top layer; the PM is arranged from top to bottom as DMDMDMDM. The layer order of the THM and FM from top to bottom are DMMDMDDM and DMDDMDMD, respectively, where M and D represent one gold and one TiO_2_ layer. All layers are 10 nm thick. The metal filling ratio for both the PM and THM is 0.5; it is 0.375 for the FM. We also investigated a modified Fibonacci multilayer (FM*), as illustrated in Figure 1d, which comprises 13.33 nm-thick metal layers and 8 nm-thick dielectric layers. This structural design tunes the metal filling ratio of the FM* to 0.5, thereby ensuring the comprehensiveness of the present study.

Cavities with a cavity height of 20 nm are also studied here, as shown in Figure 1e–h. A multilayer cavity is formed by one pair of identical multilayers, with them being mirror images with respect to an imaginary plane 10 nm from and parallel to their top surfaces. Thus, the dipole is 10 nm away from either one of the two cavity walls. A 20 nm-thick polymethyl methacrylate (PMMA, sourced from MicroChem Corp., Newton, MA, USA) layer fills the cavity between the two multilayer walls, and this PMMA layer is shown as the green region in Figure 1.

We utilize the transfer matrix method to construct all multilayers’ reflection and transmission coefficients. For TM and TE modes, the reflection coefficients are denoted as $r_{p}$ and $r_{s}$, respectively, with the transmission coefficients denoted as $t_{p}$ and $t_{s}$. These coefficients are used to study the reflectance and transmittance and their Purcell effect. In the context of semi-classical electromagnetism, the PF of a multilayer for an ideal dipole emitter polarized along *x* and *z* with unit intrinsic quantum efficiency at a distance of *d* above them is as follows [44]

(1) $$F_{x}=\frac{3}{4}Re\int_{0}^{\infty }\frac{k_{1}}{k_{z}}\frac{u}{\sqrt{\varepsilon_{1}}}\left[1+r_{s}e^{2ik_{z}d}+\frac{k_{z}^{2}}{{\varepsilon_{1}k}_{1}^{2}}\left(1-r_{p}e^{2ik_{z}d}\right)\right]du,$$

(2) $$F_{z}=\frac{3}{2}Re\int_{0}^{\infty }\frac{k_{1}}{k_{z}}{\left(\frac{u}{\sqrt{\varepsilon_{1}}}\right)}^{3}\left(1+r_{p}e^{2ik_{z}d}\right)du,$$

where $u\;=\;k_{x}/k_{1}$ is the wave-vector component parallel to the multilayer normalized by the wave-vector of light propagating inside a polymethyl methacrylate (PMMA) matrix, whose relative permittivity is $\varepsilon_{1}$. And $k_{z}\;=\;{\left(\varepsilon_{1}k_{1}^{2}\;-\;k_{x}^{2}\right)}^{1/2}$ is the wave-vector component perpendicular to the multilayer surface [44].

COMSOL 6.0 and Lumerical FDTD 2020 R2.4 are employed to investigate the dipole–multilayer coupling and the far-field emission properties of multilayers and multilayer cavities. In the COMSOL models, an electric dipolar emitter with a magnitude of 1 A·m is placed 10 nm above the surface of the multilayers, and its polarization direction is perpendicular to this surface. The electric field distribution from the dipolar emitter can be obtained in these COMSOL models. In the FDTD simulations, to study the cavity effect [45,46], the multilayer cavities are composed of two identical multilayers, which are placed symmetrically with a 20 nm gap between their top surfaces. The electric dipolar emitter with an amplitude of 1 is placed in the middle of the gap, i.e., 10 nm away from the top of each of the two multilayers. Using the built-in Lumerical scripts and integration methods, we can obtain the radiative part of the Purcell factor (Rad PF), photoluminescence at the reflection side of multilayers (PL Refl), and photoluminescence at the transmission side of multilayers (PL Trans).

In the COMSOL and FDTD simulations, the lateral dimensions of the multilayers, the thickness of the PMMA layer, and the thickness of the silica layer—along with the cavity’s lateral dimensions and its silica layer thickness—are all on a micrometer scale. Meanwhile, perfectly matched layers are implemented at the structural boundaries to avoid interference from reflected light and enable the simulation of semi-infinite periodicity.

## 3. Results and Discussion

**Figure 2:**
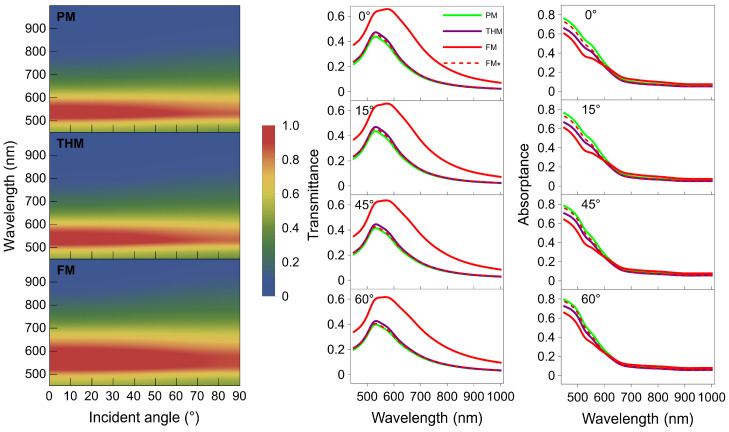
Theoretical transmittance and absorptance spectra of the PM (green), THM (purple), FM (red), and FM* (dashed red) under TM mode excitation. The left panel depicts 2-dimensional transmittance spectra as a function of incident angle and excitation wavelength; the middle and right panels selectively present transmittance and absorptance spectra at four different incident angles over a broad wavelength range.

Figure 2 shows the transmittance and absorptance spectra of the three multilayers under TM incidence calculated using the transfer matrix method. The left column shows the 2D transmittance spectra as a function of incident angle and wavelength. The transmittance minimum-to-maximum transition spectral regions (the yellow region) qualitatively indicate all multilayer band edges. The optical band edge corresponds to the spectral region with distinct transmittance variation, suggesting the potential existence of PF resonance peaks within this region. The PM and THM have band edges located around 600 nm with similar bandwidth. In contrast, the FM band-edge position is red-shifted to ~650 nm, and it covers a wider spectral range. Their transmittance resonance regions accordingly have similar spectral characteristics.

The middle and right columns showcase transmittance and absorptance spectra at four different incident angles as a function of wavelength, respectively. The solid lines are for the PM (green), THM (purple), and FM (red) with the same layer thickness, while the red dashed lines are for an adjusted FM* with the same metal filling ratio as the other two multilayers. The adjusted FM* has an 8 nm thick TiO_2_ layer and 13.33 nm gold layers. These layer thicknesses bring the FM*’s transmittance coalesced with those of the PM and THM, and keep all absorptance spectra still very close to each other. We observe in the transmittance spectra resonance features within the wavelength range of ~520–600 nm for all multilayers, while the FM transmittance resonance has a flatter and wider shape. The FM has an overall stronger transmittance due to the smaller metal filling ratio. Other than that, their spectral shapes of transmittance at different incident angles are very similar. All multilayers’ transmittance has two modes within the broad bandwidth. The two modes are associated with two PF resonances, which are discussed below, and we attribute them to the two types of coupled surface electromagnetic modes on thin metal layers [47]. The two modes arise from the coupling of surface plasmon polariton (SPP) modes at the two interfaces flanking the gold layer. The resonance wavelengths of these two modes correspond to the two aforementioned band edges.

In the shorter wavelength (<~600 nm) region at all incident angles, the magnitude of transmittance spectra follows the order with very weak differences: PM < FM* < THM < FM, while the magnitude of absorptance spectra is relatively high and has the following order: PM > FM* > THM > FM. In contrast, their absorptance magnitudes are much weaker in the wavelength range >~600 nm, with much less difference among them. These differences among all multilayers’ transmittance and absorptance spectra correlate with their radiative Purcell effects and almost identical reflectance features, which we calculated but are not shown here. We have additionally performed calculations for all multilayers under TE mode excitation (see Figure S1 in the Supplementary Information for details) and found that their spectra are highly comparable to those under the TM mode excitation reported in this work.

**Figure 3:**
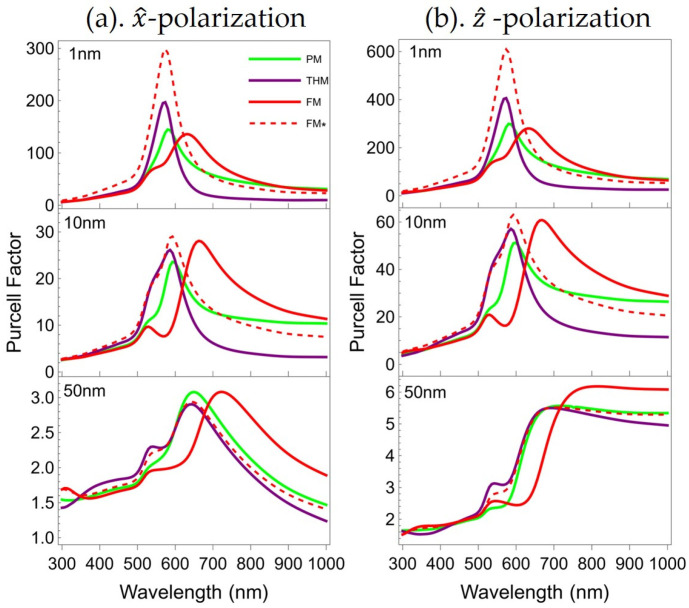
Theoretical computation of PF of a point electric dipole (**a**) parallelly and (**b**) perpendicularly polarized with respect to the PM (green), THM (purple), FM (red), and FM* (dashed red) at three different dipole heights: 1 nm (top), 10 nm (middle), and 50 nm (bottom).

The PF is used to describe the enhancement factor of the spontaneous emission rate of an atom in a resonant cavity compared to that in free space. To study the lattice-order effect on the Purcell effects of all multilayers, we use Equation (1) and (2) to calculate the PF spectra of a specific dipole, which is used to simulate an atom. This dipole is polarized both parallelly and perpendicularly to the surfaces of the multilayers, and is vertically placed at three different distances (1, 10, and 50 nm) from these surfaces. The dipole is hosted in a PMMA matrix [48], and all multilayers are on a semi-infinite silica substrate [49]. Figure 3 presents the relevant results. The PF spectra from both dipole polarizations follow the same strength order for these multilayers. The perpendicularly polarized dipole experiences stronger PFs for all dipole heights.

Previous studies reveal stronger PFs from PQCs than that of a PM counterpart, but they lack investigation into the dipole–multilayer distance effect on the PFs of various multilayers. When the metal filling ratio is the same and the dipole height is 1 nm (top row, Figure 3), the order of the PFs within the wavelength range shorter than approximately 600 nm is FM* > THM > PM for both polarizations. When the wavelength is greater than approximately 700 nm, the order of the PFs changes to PM > FM* > THM. However, as the dipole height increases, these differences gradually diminish. When the dipole height reaches 50 nm, the PFs of the three multilayers converge significantly (bottom row, Figure 3). This indicates that the Purcell effect of multilayers is dependent on the near-electric field coupling. When the dipole height is small, the near-field distribution is intense; differences in the near-field distributions of structurally distinct multilayers thereby give rise to variations in their PFs. As the dipole height increases, the near-field distribution weakens significantly, leading to a marked decrease in the PF. Furthermore, the magnitude of this weakening drowns out the differences in PFs arising from structural variations among the multilayers, ultimately leading to notable convergence of the PFs for the three multilayer types. Both the specific extent of the dipole height’s influence and the near-field distribution of the multilayers will be discussed in detail later in this text.

For multilayers with the same layer thickness, we observe a spectral dependence of the relative strength of Purcell effects of the FM and THM with respect to the PM. There are two regions in the PF spectra corresponding to the two PF resonances, which are associated with the two modes of all multilayers’ transmittance (band structure) characteristics. Within one of the two PF spectral regions, only one of the two quasiperiodic multilayers offers PF higher than that of the PM. For the two dipole polarizations and heights of 1 nm and 10 nm (the first 2 rows of Figure 3), the PF is ordered as THM ≥ PM > FM in the range of wavelengths shorter than ~600 nm. In contrast, the two quasiperiodic multilayers switch position, i.e., FM > PM > THM, in the spectral range where the wavelength is longer than ~600 nm. For instance, under the condition of *z*-polarization and for the case of 1 nm dipole height, at the PF peak wavelength of THM, the PF of THM is ~2.49 times the PF of the FM. On the other hand, at the PF peak wavelength of the FM, the PF of the FM is ~2.74 times the PF of the THM. When the dipole is not very close to the multilayers, e.g., at the dipole height of 50 nm, the PFs of the THM and PM are at the same level, while the FM has the highest PF in the long wavelength region (>~700 nm), as shown in the bottom row of both Figure 3a,b.

Figure 3 illustrates that PF resonance peaks are present within the spectral range corresponding to the two band edges. However, due to the notable difference in intensity between the two PF resonance peaks, one can lack prominence. For instance, when the dipole height equals 1 nm, only one distinct PF peak is evident for the PM, THM, and FM*. When the dipole height equals 10 nm, however, the peak shapes of the PM, THM, and FM* undergo a change at ~500 nm, confirming the presence of two types of coupled surface electromagnetic modes. When the dipole height is increased to 50 nm, the two PF peaks are clearly distinguishable.

**Figure 4:**
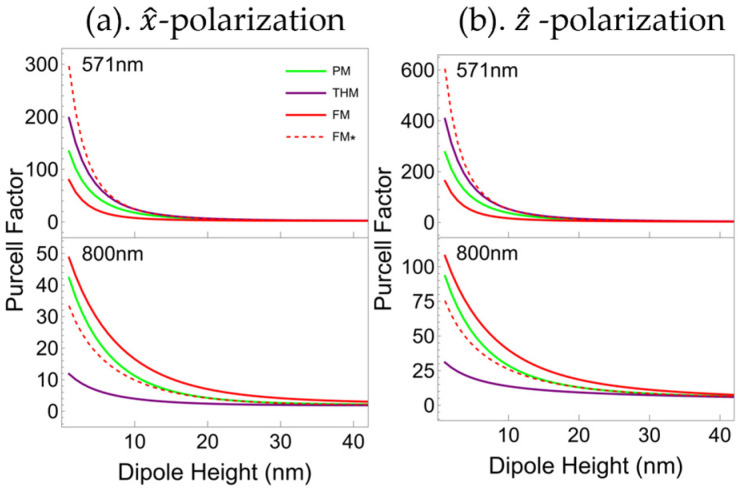
Dipole multilayer distance dependence of PF of a point electric dipole (**a**) parallelly and (**b**) perpendicularly polarized with respect to the PM (green), THM (purple), FM (red), and FM* (dashed red) at two different wavelengths: 571 nm (top) and 800 nm (bottom).

Figure 4 shows that all multilayers’ PFs are inversely proportional to the dipole–multilayer distance for both dipole polarizations. In other words, the PF of these multilayers declines rapidly as the dipole height increases. Taking a wavelength of 571 nm as a case in point, when the dipole height exceeds 10 nm, the PFs of the four multilayers diminish to a comparable magnitude. This indicates that the Purcell effect predominantly depends on a short-range electric field coupling mechanism and exhibits considerable sensitivity to dipole height. In the short wavelength range where FM* has the strongest Purcell effect at an emission wavelength of 571 nm, the rates at which the PFs weaken as the dipole–multilayer distance increases are ordered as FM* >THM > PM > FM. This order reverses to FM > PM > FM* > THM in the long wavelength range, for instance at the emission wavelength of 800 nm, where the PF of the FM is the strongest. In the whole spectral range, as the dipole–multilayer distance is comparable to the multilayer thickness, the four multilayers’ PFs are very close.

Equation (1) and (2) indicate that the dipole–multilayer distance dependence of the PFs is modulated by multilayers’ complex reflection coefficients. Among all multilayers, these coefficients contribute differently to the complex integration of PFs. This explains the difference observed in Figure 4. The contribution to the PF consists of a radiative component and a nonradiative component. The integration in the complex wavevector plane outside the light cone, i.e., the high-k or nonradiative modes, contributes the most PF of metal–dielectric multilayers [50]. We calculated the high-k part of PFs of all multilayers (see Figure S2 in Supplementary Information for details), and they are dominant parts of the PFs shown in Figure 3. This indicates that the differences among all multilayers’ PFs originate from the nonradiative modes of them.

**Figure 5:**
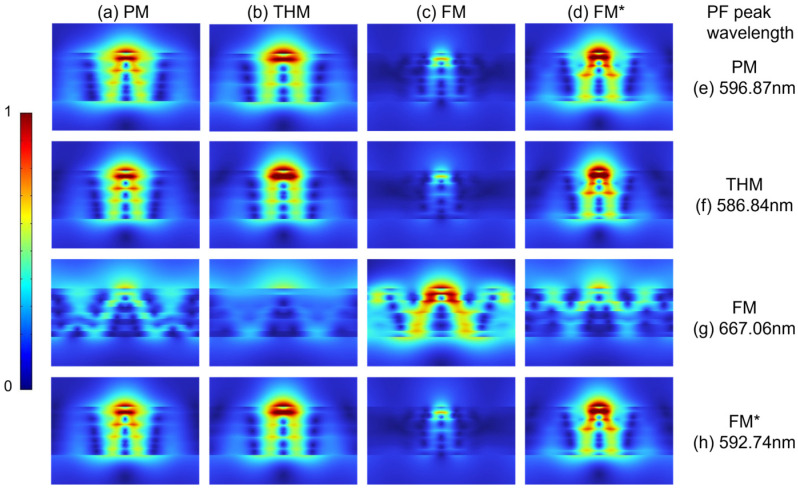
The normalized electric field magnitude distribution of a perpendicularly polarized dipolar emitter located 10 nm above all multilayers: (**a**) PM, (**b**) THM, (**c**) FM, and (**d**) FM*, corresponding to the PF excitation wavelengths of the four multilayers: (**e**) 596.87 nm, (**f**) 586.84 nm, (**g**) 667.06 nm, and (**h**) 592.74 nm.

Electric field magnitude distribution images facilitate a clearer observation of the spatial characteristics of radiative modes and—more notably—nonradiative modes. To unveil the cause for the PF differences among all multilayers, we employed the finite element analysis tool (COMSOL Multiphysics) to model the electric field magnitude distribution inside all multilayers excited from an electric dipolar emitter polarized perpendicularly to and 10 nm above all multilayers. The electric field magnitude distributions, shown in Figure 5, are simulated at four different wavelengths where each one of four multilayers’ PFs peaks, respectively. When the emission wavelengths are 596.87 nm, 586.84 nm, and 592.74 nm, the field magnitude distribution inside all multilayers tends to extend along the *z*-direction. While at the emission wavelength of 667.06 nm, where the FM’s PF peaks, the field distribution not only extends along the *z*-direction but also has a certain extension along the metal–dielectric interface (*x*-direction). However, most of the electric field is efficiently localized on or within the metal. This indicates that the majority of the energy is dissipated via metallic losses, specifically encompassing metallic ohmic losses (heat generated via electron–lattice collisions) and electron–hole excitations (energy converted into electronic excitation energy). Compared with other wavelengths, each multilayer achieves a stronger field distribution at its corresponding PF peak wavelength. A stronger field distribution implies stronger non-radiative metal loss. This corresponds to the fact that the most pronounced contributions to PFs are from the nonradiative modes associated with the electric field distribution inside the multilayers. Thus, the primary factor contributing to the differences in the PF among the multilayers is the distinct intensities of non-radiative losses within their metals, as well as the varying spatial distributions of these losses.

We then consider the Rad PFs, PL Refl, and PL Trans using the 3D full-wave electromagnetic modeling software based on the finite-difference time-domain method (Ansys Lumerical). The PL Refl and PL Trans are the dipolar emission power integrated in the reflected and transmitted side of the multilayers normalized by the total dipole power in PMMA. The power integration angle for integration at both multilayer sides is 180 degrees. The dipolar emitter is 10 nm to the multilayer top surface, and both horizontal and vertical dipole polarization are studied.

**Figure 6:**
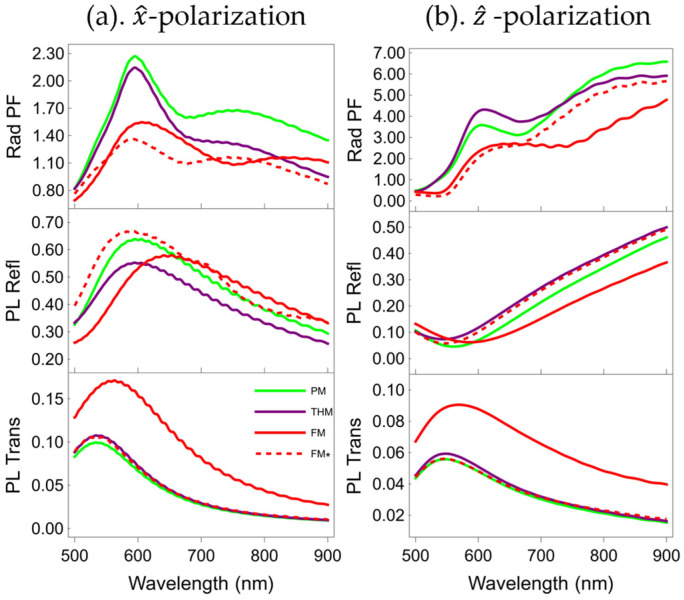
Rad PF (top), PL Refl (middle), and PL Trans (bottom) of an electric dipole at 10 nm from and (**a**) parallelly and (**b**) perpendicularly polarized with respect to the PM (green), THM (purple), FM (red), and FM* (dashed red).

Figure 6 presents the comparison of these quantities among all multilayers using the same color legends as in Figure 3. The top row of Figure 6 shows that the Rad PFs of all multilayers are much smaller than their overall PFs regardless of the electric dipole’s polarization, indicating that the PFs are mostly contributed by the non-radiative parts. Furthermore, under *z*-polarization conditions and in the long-wavelength range, a distinct cavity effect is induced inside the multilayers, as illustrated in Figure 5g. The TiO_2_ microcavities within the multilayers confine the electric field and thereby form waveguides. Specifically, this fraction of the electric field that undergoes radiative propagation within the microcavities enhances the Rad PF in the 700–900 nm range, as presented in Figure 6b.

As shown in the middle row of Figure 6, the PL Refl of the *x*-polarization is larger in the short wavelength band and gradually decreases as the wavelength increases, while the PL Refl of the *z*-polarization gradually increases.

For both polarizations, the trend and relative strength among all multilayers’ PL Trans are similar to those of transmittance spectra, which we calculated and show in Figure 2 only for the TM case. Multilayers sharing identical metal filling ratios (PM, THM, and FM*) demonstrate comparable PL Trans characteristics. In contrast, FM exhibits enhanced PL Trans owing to its lower metal filling ratio. When contrasting the two distinct polarization modes, *x*-polarization gives rise to a more pronounced peak value in PL Trans compared to *z*-polarization.

**Figure 7:**
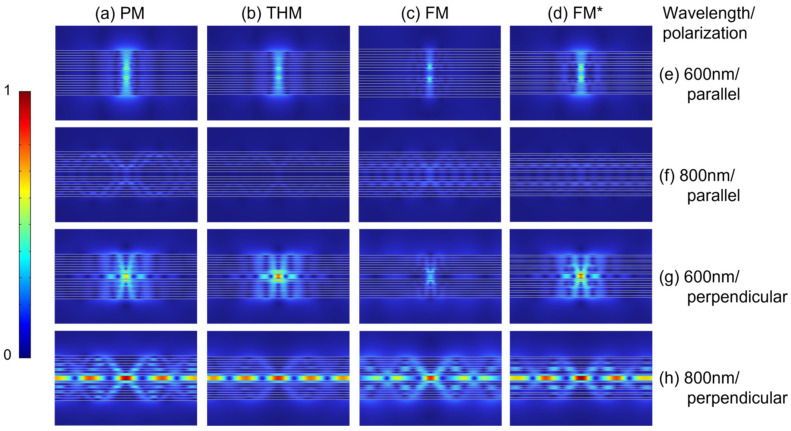
The normalized electric field magnitude distribution of a dipole emitter in the center of the cavity: (**a**) PM, (**b**) THM, (**c**) FM, and (**d**) FM*, corresponding to the wavelengths and polarization: (**e**) 600 nm/parallel, (**f**) 800 nm/parallel, (**g**) 600 nm/perpendicular, and (**h**) 800 nm/perpendicular. The lines serve as interface markers and do not exist in reality.

We also study cavities composed of these multilayers to verify that the cavity effect can enhance the differences as shown above for some excitation configurations. Figure 7 shows the normalized electric field magnitude distribution of a dipole in the middle of the cavities with emission wavelengths of 600 nm and 800 nm, respectively. When the dipole is polarized perpendicular to the cavity walls, its electric field magnitude distribution inside the cavities at 800 nm is larger than that at 600 nm, as illustrated in Figure 7g,h. Moreover, as depicted in Figure 7h, the distribution of the electric field magnitude at 800 nm wavelength is confined to the PMMA layer and exhibits a distinct lateral distribution profile. This indicates that the cavity walls, via reflection, confine a portion of the electric field within the cavity, thereby forming a standing wave along the *z*-direction. Consequently, an electric field hotspot is generated at the antinode situated at the center of symmetry. Meanwhile, the electric field propagates transversely within the cavity, and the longitudinal standing wave condition filters out and amplifies a set of degenerate modes with fixed transverse wave vectors. The dipole excites these modes, and the resonance of these modes gives rise to a periodic transverse optical field distribution at the center of symmetry. In contrast, under the parallel polarization condition, the electric field magnitude of the dipole inside cavities at 800 nm is smaller than that at 600 nm, and there is no obvious electric field magnitude distribution in the PMMA layer either, as illustrated in Figure 7e,f. We attribute this to the weak transverse emission of parallelly polarized dipoles. These findings provide support for subsequent research on the PL of cavities.

**Figure 8:**
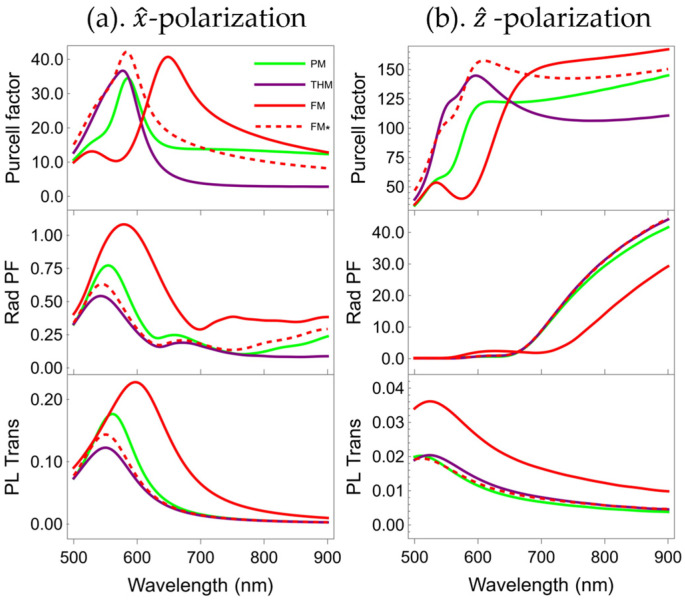
For cavities with a cavity height of 20 nm, the PF (top), Rad PF (middle), and PL Trans (bottom) under (**a**) *x*-polarization and (**b**) *z*-polarization, and for the PM (green), THM (purple), FM (red), and FM* (dashed red).

Figure 8 shows the PF, Rad PF, and PL Trans of these cavities with a dipole residing in the center. The cavity PFs are stronger than those from single multilayers for the same dipole–multilayer distance (see the 2nd row of Figure 3). For a dipolar emitter with *x*-polarization, the cavities have almost no effect on the overall PF spectral shape, and only slightly increase the PF spectral intensity compared to that of the multilayers (the difference is approximately 10). However, for the case of *z*-polarization, the multilayer cavities’ PFs are much stronger in an extended spectral range. For instance, the PF of the FM cavity is about five times that of the corresponding FM at the emission wavelength of 900 nm.

Notably, distinct polarization directions exert diametrically opposing effects on the Rad PFs when using cavity structures. The Rad PFs of the cavity for the *x*-polarization are smaller than those of the multilayers (see the top row of Figure 6 and the 2nd row of Figure 8). The Rad PF peaks of the THM and PM cavities are notably smaller than those of the multilayers, with reductions of approximately 1.5 and 1.25, respectively. In contrast, the Rad PF peak of the FM cavity is closest to that of the FM, with a difference of less than 0.5. However, for the *z*-polarization, when the wavelength is greater than 700 nm, the Rad PFs of the cavities are significantly larger than those of the multilayers (approximately 7 times). Therefore, compared with the Rad PFs of the multilayers, *x*-polarization leads to a reduction in the Rad PFs of the cavities, whereas *z*-polarization results in an enhancement of this factor for the cavities.

Through the analysis of the near-field distribution, we found that under *z*-polarization, within the 700–900 nm wavelength regime, the symmetric cavity structure induces a pronounced enhancement of the electric field magnitude at the symmetric center (i.e., the PMMA layer) and gives rise to transverse coupling modes as shown in Figure 7h. This enhancement markedly boosts Rad PF and concurrently leads to an upward trend in Rad PF across this spectral band, as illustrated in Figure 8b. The spectral range of this mode overlaps with that of the enhanced Rad PF, suggesting it as the primary driver for the Rad PF enhancement. However, via the analysis of Figure 7f and Figure 8a, it can be observed that under *x*-polarization, this mode is absent, with energy predominantly dissipated through non-radiative channels.

For *x*-polarization, the PL Trans peak values of the cavities are slightly higher than those of the multilayers. However, for *z*-polarization, the PL Trans peak values of the cavities are significantly lower than those of the multilayers. Analysis combined with the electric field magnitude distribution in Figure 7 reveals that this difference stems from the distinct radiative characteristics of the electric dipole and the cavity effect. The *x*-polarized electric dipole emits more strongly in the *z*-direction, and its electric field magnitude distribution is less prone to localization between layers, as illustrated in Figure 7e,f; furthermore, compared with a single multilayer, the additional half of the multilayer within the cavity induces reflected radiation. Therefore, the PL Trans of the cavities in Figure 8a is slightly stronger than that of the multilayers in Figure 6a. In contrast, the electric dipole of *z*-polarization has higher radiation in the *x*-direction. Following multiple reflections and interferences, the electric field magnitude distribution becomes partially localized within the cavity, as illustrated in Figure 7g,h. Consequently, the PL Trans of the cavities in Figure 8b is significantly weaker than that of the multilayers in Figure 6b.

## 4. Conclusions

We studied the Purcell effects of quasiperiodic metal–dielectric multilayers based on a one-dimensional PM, deterministic aperiodic THM, and quasiperiodic FM with the same layer thickness and FM* with the same metal filling ratio, which are composed of gold and titanium dioxide. Theoretical analysis and numerical simulations were employed to investigate their Purcell effect and PL extraction capabilities on point electric dipole emitters in the near-field region of them.

For multilayers with identical metal filling ratios, quasiperiodic multilayers exhibit a more pronounced Purcell effect over a short dipole height range. The smaller the dipole height, the more distinct this discrepancy becomes. Furthermore, a greater PF is observed when the electric dipole is polarized along the *z*-direction relative to the *x*-direction.

By comparing the dipole excitation field distributions and far-field radiative properties of these lattice systems, we found that when the PF of the FM is the most prominent among all structures, its internal dipole excitation modes extend through the entire structure in two directions. In other cases, the dipole excitation field modes within all multilayers are solely confined along the *z*-direction. Furthermore, when excited at the corresponding PF peak wavelength, the electric field intensity inside the respective multilayer forms local field hotspots. These field hotspots are related to the metal losses within the multilayers, so we attribute the observed differences in the PFs to the intensity and spatial distribution of non-radiative losses of metals inside the multilayers.

We also studied the PF and PL properties of cavities made of these multilayers. For dipoles polarized parallel to the cavity walls, most of the radiation transmits through the cavity and does not remain inside it; furthermore, compared to a single multilayer, the reflections inside the cavity can provide additional radiation along the direction perpendicular to the cavity walls. Therefore, its light extraction efficiency is higher than the corresponding efficiency of a single multilayer. For the dipole polarized perpendicular to the cavity walls, due to the cavity effect formed by the multiple reflections and interferences of the electric field at the interface, the electric field magnitude distribution is localized and enhanced within the PMMA layer. Therefore, part of the radiation cannot pass through the cavity, resulting in a lower light extraction efficiency compared to that of a single multilayer; however, this portion of radiation confined within the cavity significantly increases the Rad PF, thereby further enhancing the PF of the cavity.

This work could extend the range of extensively studied properties and applications of PQCs and pave the way towards engineering LDOS and tailoring nanoscale light–matter interactions by exploiting rich physics and phenomena originating from more complex quasicrystals with higher dimensionalities and different material disorder. The present contribution increases knowledge about the interplay of quasi-localization modes in PQCs. On the one hand, this study expands the research framework for regulating light–matter interactions via structural disorder; on the other hand, it necessitates moving beyond the constraints of conventional research directions and instead refocusing on the strong spontaneous emission enhancement achievable in complex PQCs such as cavities composed of different types of multilayers.

## Data Availability

The data that support the findings of this study are available from the corresponding author upon reasonable request.

## Supplementary Materials

The following supporting information can be downloaded at: https://www.mdpi.com/article/10.3390/nano15191502/s1, Figure S1: Theoretical transmittance and absorptance spectra of PM (green), THM (purple), FM (red) and FM* (dashed red) under TE mode excitation. The left panel depicts 2-dimensional transmittance spectra as a function of incident angle and excitation wavelength, the middle and right panel selectively presents transmittance and absorptance spectra at 4 different incident angles over a broad wavelength range. Figure S2: Theoretical computation of high-k PF of a point electric dipole (a) parallelly and (b) perpendicularly polarized with respected to PM (green), THM (purple), FM (red) and FM* (dashed red) at three different dipole heights: 1 nm (top), 10 nm (middle), and 50 nm (bottom).

## Abbreviations

The following abbreviations are used in this manuscript:

PF	Purcell Factor
PL	Photoluminescence
PQCs	Photonic Quasicrystals
LDOS	Local Density of States
PM	Periodic Multilayer
THM	Thue-Morse Multilayer
FM	Fibonacci Multilayer
FM*	Modified Fibonacci Multilayer
PMMA	Polymethyl Methacrylate
Rad PF	Radiative Part of Purcell Factor
PL Trans	Photoluminescence at the Transmission Side of Multilayers
PL Refl	Photoluminescence at the Reflection Side of Multilayers
