# Supplementary material for: Study on the Purcell Effect and Photoluminescence Properties of Gold–Titanium Dioxide Quasiperiodic Multilayers and Cavities
Source: Nanomaterials (Basel). 2025 Oct 1;15(19):1502. doi: 10.3390/nano15191502 (PMC12526272; doi:10.3390/nano15191502)
Supplement: Supplementary file 1 [file nanomaterials-15-01502-s001.zip › nanomaterials-3870827-supplementary.pdf]

Study on the Purcell Effect and Photoluminescence Properties of Gold–Titanium Dioxide Quasiperiodic Multilayers and Cavities

Guangfa He, Changjun Min, Ling Li * and Xiaocong Yuan

Nanophotonics Research Center, Institute of Microscale Optoelectronics, Shenzhen University, Shenzhen 518060, China; 2200493002@email.szu.edu.cn (G.H.); cjmin@szu.edu.cn (C.M.); xcyuan@szu.edu.cn (X.Y.)

* Correspondence: lingli@szu.edu.cn

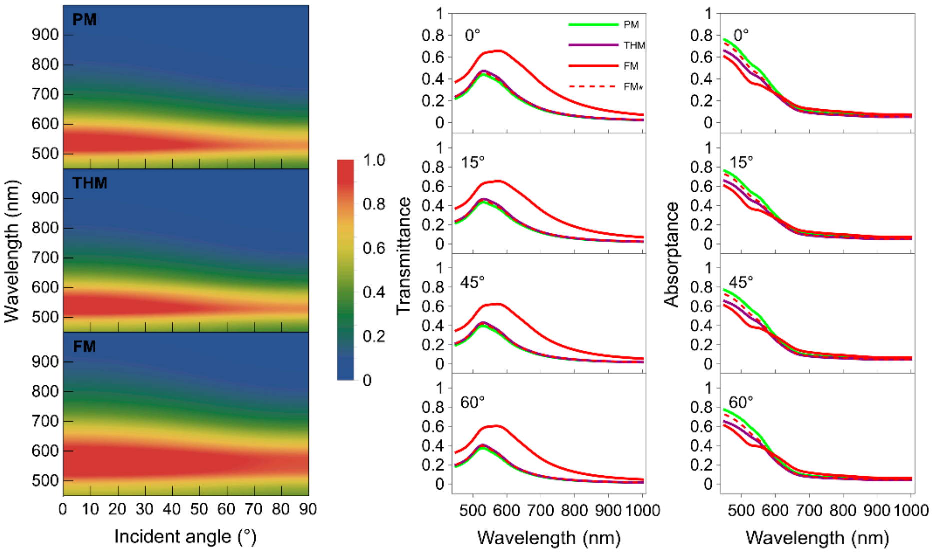

Figure S1. Theoretical transmittance and absorptance spectra of PM (green), THM (purple), FM (red) and FM* (dashed red) under TE mode excitation. The left panel depicts 2-dimensional transmittance spectra as a function of incident angle and excitation wavelength, the middle and right panel selectively presents transmittance and absorptance spectra at 4 different incident angles over a broad wavelength range.

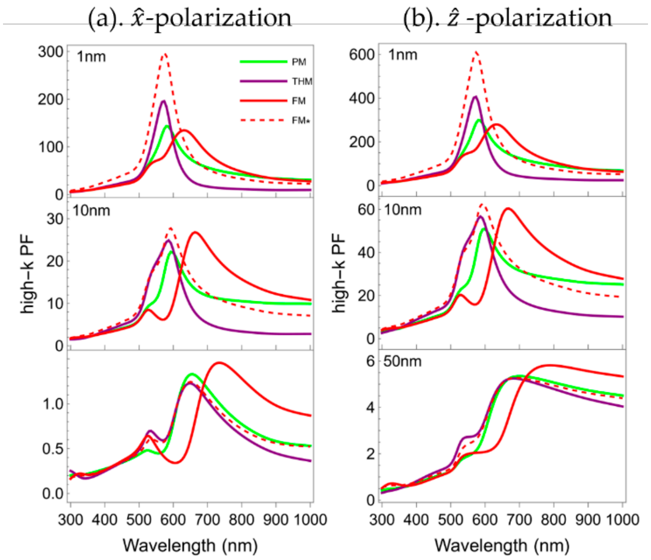

Figure S2. Theoretical computation of high-k PF of a point electric dipole (a) parallelly and (b) perpendicularly polarized with respected to PM (green), THM (purple), FM (red) and FM* (dashed red) at three different dipole heights: 1 nm (top), 10 nm (middle), and 50 nm (bottom).
